# Enhancing Critical Writing Through AI Feedback: A Randomized Control Study

**DOI:** 10.3390/bs15050600

**Published:** 2025-04-30

**Authors:** Kai Zhang

**Affiliations:** Informatization Office, Fudan University, Shanghai 200433, China; zhangkai@fudan.edu.cn

**Keywords:** artificial intelligence-generated content, critical writing, technology acceptance model, educational intervention

## Abstract

This study investigates the effectiveness of artificial intelligence-generated content (AIGC) systems on undergraduate writing development through a randomized controlled trial with 259 Chinese students. Despite promising applications of AI in educational settings, empirical evidence regarding its comparative effectiveness in writing instruction remains limited. Using a four-week intervention comparing Qwen-powered AI feedback to traditional instructor feedback, we employed difference-in-differences (DiD) analysis and structural equation modeling to examine how technology acceptance factors influence writing outcomes. Results demonstrated significant improvements in the AIGC intervention group compared to controls (β = 0.149, *p* < 0.001), with particularly strong effects on organization (β = 0.311, *p* < 0.001) and content development (β = 0.191, *p* < 0.001). Path analysis revealed that perceived usefulness fully mediated the relationship between perceived ease of use and attitudes toward the system (β = 0.326, *p* < 0.001), with attitudes strongly predicting behavioral engagement (β = 0.431, *p* < 0.001). Contrary to traditional technology acceptance models, perceived ease of use showed no direct effect on attitudes, suggesting that students prioritize functional benefits over interface simplicity in educational technology contexts. These findings contribute to an expanded technology acceptance model for educational settings while providing evidence-based guidelines for implementing AI writing assistants in higher education.

## 1. Introduction

The integration of artificial intelligence-generated content (AIGC) systems in higher education has emerged as a potential solution to address persistent challenges in developing critical writing skills (Chan & Tsi, 2023). Despite writing being a cornerstone of academic success, approximately half of Chinese university students report significant difficulties in constructing coherent arguments and evaluating evidence (Wang, 2024; Zhang & Browne, 2023). Traditional feedback mechanisms often fail to provide timely, personalized guidance due to high instructor–student ratios, creating a critical gap in formative assessment opportunities (Abulibdeh et al., 2024).

Effective feedback is crucial for writing development, with meta-analyses demonstrating that detailed, timely response to student work yields significant improvements in composition quality (McCarthy et al., 2022; Steiss et al., 2024). However, traditional instructor feedback mechanisms often fail to provide timely, personalized guidance due to high instructor–student ratios, creating a critical gap in formative assessment opportunities. This gap has motivated exploration of alternative feedback sources, including peer assessment, automated writing evaluation systems, and more recently, AI-generated feedback (Banihashem et al., 2024).

Research comparing AI-generated and instructor feedback has yielded mixed results. Early automated systems showed modest effects primarily on mechanical aspects of writing (Nunes et al., n.d.; Shi & Aryadoust, 2024), while more recent large language model implementations demonstrate improvements in higher-order skills like argumentation and evidence use. Studies found AI feedback was perceived as more specific but less motivating than instructor comments (Gan et al., 2021), while comparative study showed equivalent writing gains between conditions but different improvement trajectories. These findings suggest potential complementarity between feedback types, but do not account for individual differences that might moderate effectiveness.

The technology acceptance model (TAM) provides a theoretical framework for understanding how psychological factors influence the effectiveness of educational technologies (Granić & Marangunić, 2019). Recent meta-analyses reveal moderate effect sizes for TAM in predicting technology adoption but consistently highlight unexplained variance (35–42%) attributable to contextual moderators (Al-Adwan et al., 2023; Salloum, 2018; Tawafak et al., 2023).

Despite the growing body of research on AI feedback in writing instruction, two significant research gaps persist. First, randomized controlled trials comparing AIGC writing systems with traditional instruction remain scarce in the literature. Most studies exhibit methodological limitations including self-selection bias and inconsistent feedback conditions (Nissen et al., 2025; Zhu et al., 2022). The few existing experimental studies rarely control for feedback timing or structural equivalence across conditions, preventing conclusive determinations about the causal impact of AI feedback on writing outcomes (Banihashem et al., 2024; Song & Song, 2023; Wang, 2024). This methodological gap significantly limits our understanding of AIGC’s true effectiveness compared to traditional approaches.

Second, research has insufficiently explored the mechanisms influencing AIGC adoption and effectiveness. While the technology acceptance model (Granić, 2022; Granić & Marangunić, 2019; Marangunić & Granić, 2015) has been widely applied to educational technologies, significant unexplained variance in adoption patterns suggests important moderators remain unidentified. Several studies identified perceived usefulness as the primary determinant of technology acceptance (Pan et al., 2024), while others found perceived ease of use to be more critical for educational technologies (Malmous & Zaidoune, 2025; Tan, 2024). This inconsistency highlights a significant gap in understanding which TAM constructs are most influential in different educational contexts.

Addressing these gaps is important for several reasons. First, as educational institutions increasingly deploy AI writing assistants, empirically validated implementation guidelines are needed to maximize their effectiveness. Second, understanding how individual differences interact with feedback modalities enables more personalized pedagogical approaches. Finally, from a theoretical perspective, investigating moderators of technology acceptance addresses a significant blind spot in educational technology frameworks, potentially resolving contradictory findings in previous literature.

This study addresses these gaps through a randomized controlled trial examining the effects of a Qwen-powered AIGC writing intervention on undergraduate students’ critical writing performance. Additionally, we investigate how the technology acceptance model factors influence writing outcomes, with a focus on perceived usefulness, ease of use, and attitude toward system use. Two hypotheses guide our investigation:

(1) The AIGC intervention will significantly improve critical writing scores compared to traditional instruction, with effects manifested across multiple dimensions including content development, organizational structure, and language usage.
(2) Technology acceptance factors will influence writing outcomes through a sequential mediation pathway, whereby perceived ease of use will enhance perceived usefulness, which will positively affect attitudes toward system use, ultimately leading to increased behavioral engagement and improved writing performance.

By employing difference-in-differences analysis and structural equation modeling, this research advances theoretical understanding of stress–technology interactions while providing practical insights for adaptive AIGC system design.

## 2. Method

### 2.1. Participants

This study was conducted at a university in China following ethical approval from the institutional review board (FDU-2024-041, 23 April 2024). According to the empirical formula (10 times the index number) and the efficacy analysis assuming a moderate-intensity intervention effect, this study requires a minimum sample of 100 people (Christopher Westland, 2010). Participants were voluntarily enrolled after providing written informed consent and were randomly assigned to either the intervention group (n = 130) or the control group (n = 129) using a 1:1 allocation ratio stratified by gender and academic year. Demographic characteristics, including gender, age, and household income, were collected at baseline. All participants completed a one-month critical writing course, with attendance rates exceeding 95% in both groups.

This study employed a pre–post, double-blind randomized controlled trial design to evaluate the effectiveness of AI-generated writing feedback compared to traditional instructor feedback. After baseline assessments (writing task 1 and psychometric measures), participants were randomly assigned to either the AI feedback (intervention) or human feedback (control) condition. Both groups completed identical writing tasks and received feedback following the same timeline over the four-week intervention period. Post-intervention assessments included a second writing task and follow-up psychometric measures.

This study recruited 412 initial applicants through campus-wide postings and digital announcements targeting third-year undergraduates enrolled in academic writing courses during May 2024. Participants were recruited from five academic faculties: Humanities (34.7%), Social Sciences (27.4%), Natural Sciences (18.5%), Engineering (11.2%), and Business (8.1%). Recruitment occurred through campus-wide postings, departmental emails, and digital announcements on the university’s learning management system. Recruitment materials described a study on “writing improvement methods” without specifying AI components to avoid selection bias.

Eligibility screening applied four criteria: (1) native Mandarin proficiency, (2) intermediate English competency (CET-4 scores 425–550), (3) no prior AI writing tool experience, and (4) baseline Perceived Stress Scale (PSS) scores within ±1.5 SD of population norms (18–32). The final sample of 259 eligible participants underwent randomization using computer randomization generation. Baseline equivalence testing confirmed no significant group differences in demographic variables, initial writing score, or stress levels. Double-blind procedures ensured instructors graded all submissions without group awareness.

### 2.2. Intervention

The four-week intervention employed a counterbalanced design with parallel feedback systems. Participants in the AIGC condition received automated critiques through a Qwen-powered platform that analyzed drafts within 12–24 h of submission. The system generated structured feedback focusing on three core elements: argument coherence (through logical flow mapping), evidence quality (via source credibility scoring), and rhetorical effectiveness (using persuasion metrics). To maintain pedagogical integrity, the AI was constrained to diagnostic comments and Socratic questioning—explicitly prohibited from content generation—with fixed response lengths (150–200 words) and maximum three feedback iterations per draft.

For the AIGC intervention, we used a fine-tuned version of the Qwen-7B-Chat model customized for academic writing evaluation. The model was trained on 12,000 annotated college-level essays with instructor feedback and specialized prompts for critical analysis. The AI system was designed to identify logical fallacies, evidence gaps, and opportunities for deeper analysis.

The control group received human feedback from two trained instructors with over five years of writing pedagogy experience, using identical rubrics and response timelines. Both groups completed matched writing tasks (two 800-word essays) under equivalent conditions: shared learning management system access for course materials, synchronized peer review sessions, and identical deadlines. Contamination prevention measures included segregated LMS instances, IP-based access controls, and automated usage monitoring. To ensure feedback comparability, instructors received 20 h of training on the feedback protocol and were provided with standardized feedback templates aligned with the AI system’s output structure. Instructors were instructed to provide the same quantity of feedback points (5–7 per submission) with similar word counts (150–200 words). Inter-rater consistency between instructors was established through pilot feedback sessions (κ = 0.79 for feedback categorization).

Several measures ensured intervention fidelity. First, all feedback (both AI and human) was reviewed by a research assistant not involved in the study design to verify adherence to the feedback protocol. Second, we analyzed feedback samples from both conditions (20% random selection) to confirm comparable feedback characteristics including word count, focus areas, suggestion specificity, and tone. Third, we monitored participant engagement through system logs to ensure comparable time spent reviewing feedback across conditions. Fourth, participants were not informed about their condition assignment to prevent expectancy effects.

### 2.3. Measurements

Writing performance was assessed through two 800-word argumentative essays completed at pre-intervention (Week 1) and post-intervention (Week 4). Essay topics were counterbalanced across participants to control for potential topic effects. The pre-intervention topic asked students to “Discuss the benefits and challenges of implementing artificial intelligence in healthcare systems,” while the post-intervention topic asked students to “Evaluate the ethical implications of using genetic engineering in agriculture”. Topics were selected based on pilot testing with a separate sample (n = 42) to ensure comparable difficulty levels.

Writing performance was assessed using a comprehensive, multi-dimensional rubric across five key domains. Overall writing score (0–100 points) represented the cumulative evaluation of students’ writing proficiency across all assessment dimensions. Content (0–25 points) measured the quality of ideas, argumentative strength, and evidence integration within written compositions. Organization (0–25 points) evaluated structural coherence, logical progression, and effective paragraph development. Language usage (0–25 points) assessed grammatical accuracy, vocabulary richness, and stylistic appropriateness. Writing motivation (0–25 points) captured students’ engagement, commitment, and attitudes toward the writing process (Song & Song, 2023). All writing samples were independently evaluated by two raters blind to experimental condition and assessment time, with excellent inter-rater reliability (ICC = 0.88 for pre-test; ICC = 0.91 for post-test). Any scoring discrepancies exceeding 10 points were resolved through a third-rater discussion to ensure assessment reliability and validity.

Technology acceptance was measured using the 12-item technology acceptance model (TAM) questionnaire ((Davis, 1989); α = 0.72 in current study, comparable to historical α = 0.78–0.85), assessing perceived usefulness and ease of use. The questionnaire included four items measuring perceived usefulness (e.g., “Using the feedback system improves my writing performance”; α = 0.79), four items measuring perceived ease of use (e.g., “Learning to use the feedback system was easy for me”; α = 0.76), and four items measuring attitude toward using the system (e.g., “Using the feedback system is a good idea”; α = 0.81). Items were rated on a 7-point Likert scale from 1 (strongly disagree) to 7 (strongly agree). Confirmatory factor analysis supported the three-factor structure (CFI = 0.94, RMSEA = 0.058, SRMR = 0.062).

Perceived stress was quantified through the 10-item Chinese version of the Perceived Stress Scale ((Lu et al., 2017); α = 0.81). AI engagement was objectively tracked via system-logged token counts, reflecting feedback iteration frequency and depth. This scale measures the degree to which situations in one’s life are appraised as stressful, with items such as “In the last month, how often have you felt nervous and stressed?” and “In the last month, how often have you felt that you were unable to control the important things in your life?” Items are rated on a 5-point scale from 0 (never) to 4 (very often), with higher scores indicating greater perceived stress. The Chinese PSS has demonstrated good reliability and validity in university student populations (Lu et al., 2017). In our sample, scores ranged from 11 to 36 (M = 24.79, SD = 5.55).

Confirmatory factor analysis in Table 1 established adequate measurement properties for the TAM constructs. All standardized loadings exceeded 0.60 (M = 0.67, SD = 0.06), with perceived usefulness (PU) items demonstrating the strongest loadings (range = 0.68–0.75). The attitude (AT) factor showed particularly robust indicators (loadings = 0.69–0.72), while perceived ease of use (PEOU) items exhibited slightly lower but acceptable loadings (0.60–0.66). All factors achieved good reliability (PU: α = 0.78; PEOU: α = 0.71; AT: α = 0.75), with composite reliabilities ranging from 0.81 to 0.86. Modification indices suggested no substantial cross-loadings or correlated errors.

### 2.4. Statistical Analysis

A difference-in-differences (DiD) framework estimated intervention effects on writing scores, controlling for baseline covariates (gender, age, household income). The effect of intervention is captured through the coefficient of the $\left({\mathrm{Treatment}}_{i}\times {\mathrm{Time}}_{t}\right)$ interaction term (δ) in the following generalized specification

$$Y_{it}=\alpha +\beta \cdot {\mathrm{Treatment}}_{i}+\gamma \cdot {\mathrm{Time}}_{t}+\delta \cdot \left({\mathrm{Treatment}}_{i}\times {\mathrm{Time}}_{t}\right)+\varepsilon_{it}$$

where $Y_{it}$ = writing performance score for individual i at time t, ${\mathrm{Treatment}}_{i}$ = binary treatment indicator (1 = treatment group, 0 = control group), ${\mathrm{Time}}_{t}$ = binary temporal indicator (1 = post-intervention, 0 = pre-intervention), and $\varepsilon_{it}$ = error term.

The parameter δ formally represents the Average Treatment Effect on the Treated (ATT) and can be decomposed as:

$$\begin{array}{l} \hat{\delta }=ATT=\left[E\left(Y_{it}|{\mathrm{Treatment}}_{i}=1,{\mathrm{Time}}_{t}=1\right)-E\left(Y_{it}|{\mathrm{Treatment}}_{i}=1,{\mathrm{Time}}_{t}=0\right)\right]\\ \;\;\;\;\;\;-\left[E\left(Y_{it}|{\mathrm{Treatment}}_{i}=0,{\mathrm{Time}}_{t}=1\right)-E\left(Y_{it}|{\mathrm{Treatment}}_{i}=0,{\mathrm{Time}}_{t}=0\right)\right] \end{array}$$

For the structural equation modeling (SEM) analysis, maximum likelihood estimation in lavaan evaluated direct and indirect pathways between AI engagement (token count), TAM factors, stress levels, and writing outcomes. Model fit was assessed using CFI (>0.95), TLI (>0.95), RMSEA (<0.06), and SRMR (<0.08) (Schreiber, 2017).

## 3. Results

### 3.1. Descriptive Statistics

The final analytic sample included 259 participants (intervention: n = 125; control: n = 134, detailed in Table 2) with comparable baseline characteristics across groups. Randomization successfully balanced demographic variables, as evidenced by non-significant between-group differences in gender proportion (control = 49%, intervention = 55%; *p* = 0.283), age (control = 22.24, SD = 1.10; intervention = 22.01, SD = 1.18; *p* = 0.108), household income (control = CNY 81,914, SD = 32,716; intervention = CNY 81,282, SD = 37,428; *p* = 0.885), and perceived stress levels (control = 24.60, SD = 5.56; intervention = 24.97, SD = 5.54; *p* = 0.591).

### 3.2. Difference-in-Difference Analysis

The difference-in-differences (DiD) framework was implemented as a single comprehensive analytical procedure, employing a standardized estimation methodology across all outcome variables. This approach enables direct estimation of the Average Treatment Effect on the Treated (ATT) through the coefficient of the Treatment × Time interaction term.

As presented in Table 3, the DiD analysis revealed that the AIGC intervention significantly improved overall writing scores compared to the control condition (β = 0.149, SE = 0.044, *p* < 0.001). This indicates that students in the treatment group demonstrated a 0.149 standard deviation greater improvement than those in the control group after adjusting for relevant covariates. The effect remained consistent and statistically significant across all writing dimensions, with particularly pronounced effects on organization (β = 0.311, SE = 0.048, *p* < 0.001) and content (β = 0.191, SE = 0.045, *p* < 0.001) scores.

Examining the component scores provides deeper insight into the specific writing domains most responsive to the AIGC intervention. The strongest effect was observed in organization scores, where the treatment group showed a 0.311 standard deviation greater improvement compared to controls. Content scores showed the second largest improvement (0.191 SD), while language usage (β = 0.070, SE = 0.043, *p* < 0.05) and writing motivation (β = 0.077, SE = 0.044, *p* < 0.05) demonstrated more modest yet still significant enhancements.

The main effects of Treatment and Time further illustrate important patterns. The significant Time coefficients across dimensions (ranging from β = 0.083 to β = 0.274) indicate general improvement in writing skills over the intervention period regardless of group assignment. Meanwhile, the significant Treatment coefficients for content, organization, and writing motivation suggest pre-existing differences between groups despite randomization, which our DiD design appropriately accounts for by focusing on differential change rather than absolute differences.

Among the covariates, age emerged as the strongest predictor of writing performance across all dimensions (β = 0.251 to β = 0.380, all *p* < 0.001), followed by household income (β = 0.169 to β = 0.242, all *p* < 0.001). Sex showed significant effects on overall writing, content, organization, and language usage, with female students generally performing better than males. The full model demonstrated good explanatory power, accounting for 22.7% of variance in overall writing scores and between 14.1% and 23.5% for specific dimensions.

### 3.3. Path Analysis

**Figure 1:**
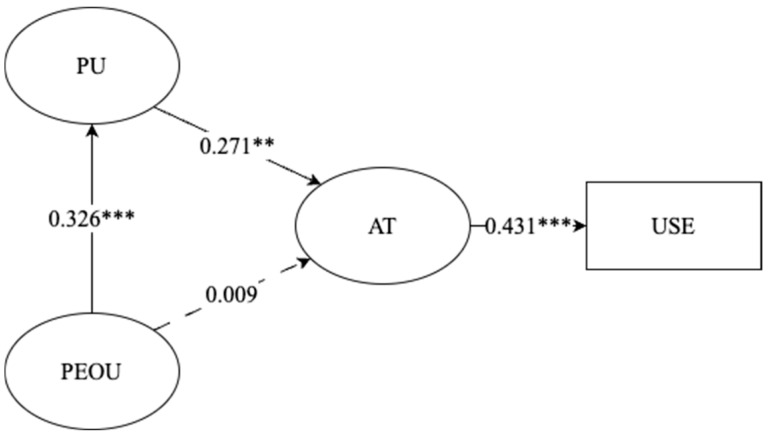
Path analysis model illustrating the relationships between perceived ease of use (PEOU), perceived usefulness (PU); attitude toward use (AT), and actual system usage (USE) with standardized path coefficients. Note. PEOU = perceived ease of use; PU = perceived usefulness; AT = attitude toward use; USE = system usage (token usage). Solid arrows indicate statistically significant paths, while the dashed arrow represents a non-significant relationship. ** *p* < 0.01; *** *p* < 0.001.

The results of the model are showed in Table 4, with a chi-square of 24.803, *p* = 0.814, CFI = 0.99, TLI = 0.98, RMSEA = 0.03, and SRMR = 0.04. Perceived usefulness fully mediated the relationship between PEOU and AT (β = 0.326, *p* < 0.001), with PU directly predicting positive attitudes (β = 0.271, *p* = 0.002). Behavioral engagement with the AI system (token usage) was strongly predicted by AT (β = 0.431, *p* < 0.001), accounting for 18.6% of usage variance. Contrary to hypotheses, PEOU showed no direct effect on AT (β = −0.009, *p* = 0.922). The model explained 31.2% of variance in final writing scores through these mediated pathways (Figure 1).

## 4. Discussion

The current study extends our understanding of AI-feedback writing interventions by elucidating the dual role of perceived stress in moderating both technological engagement and pedagogical outcomes. Our findings substantiate the central hypothesis that AIGC systems can enhance critical writing performance (β = 0.149, *p* < 0.001). The difference-in-differences analysis demonstrated that students receiving AI-generated feedback showed significant improvements across various writing dimensions compared to traditional instruction, with particularly strong effects on organization and content development. These results challenge conventional approaches to writing instruction by highlighting how technological interventions can create more personalized, timely feedback loops that facilitate deeper engagement with the writing process. The technology acceptance model analysis further illuminates the mechanisms underlying these improvements, suggesting that perceived usefulness and positive attitudes toward AI systems serve as critical mediators of behavioral engagement and subsequent writing outcomes.

### 4.1. Verification of Hypotheses

The first hypothesis regarding AIGC’s efficacy received strong support, with intervention participants demonstrating significant improvements in writing scores compared to the control group. The difference-in-differences analysis revealed a significant interaction effect (Treatment × Time) for overall writing performance (β = 0.149, *p* < 0.001), indicating that the AIGC intervention enhanced students’ writing skills beyond what would be expected from traditional instruction alone. This effect was consistent across multiple writing dimensions, with particularly strong improvements observed in organization (β = 0.311, *p* < 0.001) and content development (β = 0.191, *p* < 0.001), while more modest yet still significant gains were seen in language usage (β = 0.070, *p* < 0.05) and writing motivation (β = 0.077, *p* < 0.05).

These improvements suggests that the AIGC intervention facilitated systemic rather than superficial learning gains. It is notable that organization showed the strongest response to the intervention, suggesting that AI-generated feedback may be particularly effective in helping students structure their arguments coherently. Content improvements indicate that students were able to develop more substantive and well-supported arguments, while the gains in language usage reflect enhanced technical writing skills.

Several demographic covariates emerged as significant predictors of writing performance. Age consistently demonstrated the strongest relationship with writing outcomes across all dimensions (β ranging from 0.251 to 0.380, all *p* < 0.001), suggesting that developmental factors play an important role in writing proficiency. Household income also showed consistent positive associations with writing performance (β ranging from 0.169 to 0.242, all *p* < 0.001), highlighting the influence of socioeconomic factors on academic achievement. Sex differences were evident in most writing dimensions, with female students generally outperforming male students.

The second hypothesis concerning technology acceptance factors was also supported by our path analysis results. As predicted, perceived ease of use significantly enhanced perceived usefulness (β = 0.326, *p* < 0.001), which in turn positively influenced attitudes toward system use (β = 0.271, *p* < 0.01). Contrary to traditional technology acceptance models, we found that perceived usefulness fully mediated the relationship between ease of use and attitudes, with no significant direct path from ease of use to attitudes (β = −0.009, *p* = 0.922). This suggests that in educational technology contexts, students prioritize functionality and effectiveness over interface simplicity.

Furthermore, positive attitudes toward the system strongly predicted behavioral engagement (β = 0.431, *p* < 0.001), which subsequently contributed to improved writing outcomes. This sequential mediation pathway explains how technology acceptance factors translated into tangible educational benefits, supporting our hypothesized mechanism of action for the AIGC intervention’s effectiveness.

### 4.2. Theoretical Implications

The findings of this study offer several important theoretical contributions to both educational technology and writing pedagogy literature. First, our results provide empirical support for an expanded technology acceptance model (TAM) in educational contexts by demonstrating that the traditional relationship between perceived ease of use and attitudes is fully mediated by perceived usefulness. This challenges the conventional TAM assumption (Granić & Marangunić, 2019; Marangunić & Granić, 2015; Nnaji et al., 2023; Pan et al., 2024) that ease of use directly influences attitudes independent of utility perceptions, suggesting instead that students prioritize functional benefits over interface simplicity when evaluating educational technologies (Luo, 2024; Vlasenko et al., 2023). This reconceptualization helps explain previous inconsistencies in TAM applications to educational settings and advances a more nuanced understanding of technology adoption in learning environments.

Second, our findings contribute to writing pedagogy theories by demonstrating differential effects of AI feedback across writing dimensions. The pronounced improvements in organization (β = 0.311) compared to more modest gains in language usage (β = 0.070) suggest that higher-order cognitive aspects of writing may be more responsive to structured, analytical feedback than mechanical elements. This pattern challenges traditional writing instruction hierarchies that often prioritize grammatical correctness before addressing structural coherence (Siekmann et al., 2022) and instead supports a more integrated approach where organizational scaffolding provides a foundation for content development.

Finally, our results contribute to the emerging theoretical framework for AI-human educational partnerships by demonstrating that AI feedback can complement rather than replace traditional instruction. The improvements observed across writing dimensions indicate that AI systems may effectively address specific bottlenecks—particularly in areas requiring iterative feedback, such as organization contents—without diminishing the value of human instruction in motivational and affective domains. This supports a synergistic rather than substitutive theoretical model for AI integration in education.

### 4.3. Practical Applications

The findings from this study yield several practical applications for educational institutions, instructors, and educational technology developers. First, the significant writing improvements observed in the AIGC intervention group provide compelling evidence for integrating AI writing assistants into undergraduate curricula, particularly for courses with large enrollment where personalized instructor feedback is constrained. Universities could implement similar systems as supplementary resources that provide formative feedback between formal instructor evaluations, creating a multi-tiered feedback ecosystem that enhances the frequency and specificity of writing guidance.

Second, the demographic patterns identified in our analysis highlight the importance of equity considerations in AI implementation. The significant relationships between writing performance and socioeconomic indicators (household income: β = 0.169–0.242) suggest that AI writing tools could serve as equalizers by providing high-quality feedback to students with limited access to educational resources. Institutions should consider subsidizing access to these technologies for lower-income students to prevent digital divides from exacerbating existing educational inequalities.

Finally, our findings suggest specific training approaches for maximizing AI writing assistant effectiveness. The behavioral engagement patterns observed in our study indicate that students benefit from explicit instruction in feedback utilization strategies, particularly for translating AI recommendations into substantive revisions. Faculty development programs should incorporate modules on AI-augmented writing pedagogy that prepare instructors to complement algorithmic feedback with human guidance on motivation and writing process management.

### 4.4. Limitations and Future Directions

While this study advances our understanding of AI-enhanced writing instruction, several limitations should be acknowledged. First, the intervention duration of four weeks, while sufficient to demonstrate immediate effects, precludes assessment of long-term retention and transfer of writing skills. Future research should employ longitudinal designs spanning multiple semesters to evaluate whether improvements persist beyond the intervention period and transfer to different writing contexts or disciplines.

Second, our sample was limited to undergraduate students at a single Chinese university, potentially restricting generalizability across educational levels and cultural contexts. The predominantly upper-middle socioeconomic profile of participants (mean household income CNY 81,600) may have influenced technology acceptance patterns in ways that would not apply to more diverse populations. Future studies should include multi-institutional samples with greater demographic variation to establish boundary conditions for intervention effectiveness.

Third, the current study focused exclusively on argumentative essays, which may not represent the full spectrum of academic writing genres. Different writing tasks (e.g., narrative, technical, reflective) may respond differently to AI feedback based on their structural and rhetorical requirements. Future research should examine how genre-specific features moderate AIGC effectiveness across diverse writing assignments.

### 4.5. Conclusions

This study provides empirical evidence that AI-generated content systems can significantly enhance undergraduate students’ critical writing skills when implemented as structured feedback interventions. The difference-in-differences analysis demonstrated substantive improvements across multiple writing dimensions, with particularly strong effects on organizational structure and content development. These findings suggest that AIGC systems can address persistent challenges in writing instruction by providing timely, detailed feedback that complements traditional teaching approaches.

The path analysis further illuminated the mechanisms underlying these improvements, revealing that perceived usefulness serves as the primary driver of system adoption and subsequent engagement. This challenges conventional technology acceptance models by demonstrating that ease of use operates primarily through usefulness perceptions rather than directly influencing attitudes. This reconceptualization helps explain previous inconsistencies in educational technology adoption patterns and provides a more nuanced framework for understanding how students evaluate and engage with AI learning tools.

From a practical perspective, this study offers evidence-based guidelines for designing and implementing AI writing assistants in higher education. The differential effects observed across writing dimensions suggest that these systems should prioritize feedback on higher-order aspects like organization and argumentation, while the demographic patterns highlight opportunities for using AIGC to address educational inequities. As AI technologies continue to evolve, their thoughtful integration into writing pedagogy offers promising avenues for enhancing instruction quality and accessibility.

In conclusion, this research advances both theoretical understanding and practical application of AI-enhanced writing instruction while identifying important directions for future investigation. By demonstrating how technology acceptance factors influence writing outcomes and elucidating the specific mechanisms of AIGC effectiveness, this study contributes to an emerging framework for human-AI educational partnerships that leverages technological capabilities while preserving essential human elements of writing instruction.

## Figures and Tables

**Table 1:** CFA Factor Loadings.

Latent Factor	Indicator	B	95% CI	sig	SE	z
AT	at1	0.692	0.594–0.791	***	0.05	13.81
AT	at2	0.623	0.520–0.726	***	0.053	11.855
AT	at3	0.719	0.622–0.816	***	0.049	14.537
PEOU	peou1	0.662	0.538–0.785	***	0.063	10.503
PEOU	peou2	0.612	0.489–0.734	***	0.063	9.763
PEOU	peou3	0.601	0.479–0.724	***	0.063	9.604
PU	pu1	0.718	0.627–0.809	***	0.046	15.507
PU	pu2	0.748	0.658–0.837	***	0.046	16.417
PU	pu3	0.68	0.587–0.773	***	0.048	14.305

AT = attitude toward use; PEOU = perceived ease of use; PU = perceived usefulness. *** *p* < 0.001.

**Table 2:** Sample Characteristics.

	Control	Treatment	*p*-Value
Observation	134	125	
Gender (%, 1 = male)	0.49	0.55	0.283
Age (mean (SD))	22.24 (1.10)	22.01 (1.18)	0.108
PSS (mean (SD))	24.60 (5.56)	24.97 (5.54)	0.591
Household income (mean (SD))	81,914.42 (32,715.51)	81,282.33 (37,428.19)	0.885

**Table 3:** DiD Results.

	Overall Writing Score	Content Score	Organization Score	Language Usage Score	Writing Motivation Score
	(1)	(2)	(3)	(4)	(5)
Treatment × Time	0.149 ***	0.191 ***	0.311 ***	0.070 *	0.077 *
	(0.044)	(0.045)	(0.048)	(0.043)	(0.044)
Treatment	0.064	0.079 *	0.186 ***	0.065	0.155 ***
	(0.044)	(0.046)	(0.044)	(0.042)	(0.044)
Time	0.274 ***	0.239 ***	0.083 *	0.165 ***	0.098 **
	(0.045)	(0.046)	(0.046)	(0.044)	(0.045)
Age	0.369 ***	0.339 ***	0.328 ***	0.251 ***	0.380 ***
	(0.045)	(0.046)	(0.044)	(0.044)	(0.047)
Sex	0.100 **	0.152 ***	0.114 **	0.142 ***	0.063
	(0.046)	(0.045)	(0.046)	(0.045)	(0.045)
Income	0.169 ***	0.242 ***	0.227 ***	0.218 ***	0.232 ***
	(0.046)	(0.048)	(0.044)	(0.044)	(0.045)
PSS	0.091 **	0.078 *	0.037	0.053	0.008
	(0.046)	(0.046)	(0.042)	(0.045)	(0.045)
Constant	0.067	0.062	0.004	−0.017	−0.050
	(0.045)	(0.045)	(0.045)	(0.044)	(0.045)
Observations	514	514	514	514	514
R^2^	0.227	0.220	0.235	0.141	0.176
Adjusted R^2^	0.217	0.209	0.224	0.129	0.165

Note. * *p* < 0.05; ** *p* < 0.01; *** *p* < 0.001.

**Table 4:** Path Analysis.

DV	Predictor	B	95% CI	sig	SE	z
AT	PEOU	−0.009	−0.197–0.178		0.096	−0.098
AT	PU	0.271	0.101–0.442	**	0.087	3.116
PU	PEOU	0.326	0.165–0.487	***	0.082	3.973
USE	AT	0.431	0.310–0.553	***	0.062	6.962

Note. PEOU = perceived ease of use; PU = perceived usefulness; AT = attitude toward use; USE = system usage (token usage). ** *p* < 0.01; *** *p* < 0.001.

## Data Availability

Data cannot be shared publicly because of Ethic regulation. Data are available from the corresponding author for researchers who meet the criteria for access to de-identified data.
